# Design and evaluation of Lumefantrine – Oleic acid self nanoemulsifying ionic complex for enhanced dissolution

**DOI:** 10.1186/2008-2231-21-27

**Published:** 2013-03-25

**Authors:** Ketan Patel, Vidur Sarma, Pradeep Vavia

**Affiliations:** 1 Center for Novel Drug Delivery Systems, Department of Pharmaceutical Sciences and Technology, Institute of Chemical Technology, University under Section 3 of UGC Act – 1956, Elite Status and Center of Excellence – Govt. of Maharashtra, TEQIP Phase II Funded, N. P. Marg, Matunga (E), Mumbai, 400 019, India

## Abstract

**Background:**

Lumefantrine, an antimalarial molecule has very low and variable bioavailability owing to its extremely poor solubility in water. It is recommended to be taken with milk to enhance its solubility and bioavailability. The aim of present study was to develop a Self Nanoemulsifying Delivery system (SNEDs) of lumefantrine (LF) to achieve rapid and complete dissolution independent of food-fat and surfactant in dissolution media.

**Methods:**

Solubility of LF in oil, co-solvent/co-surfactant and surfactant solution and emulsification efficiency of surfactant were analyzed to optimize the LF loaded self nanoemulsifying preconcentrate. Effect of LF-oleic acid complexation on emulsification, droplet size, zeta potential and dissolution were investigated. Effect of milk concentration and fat content on saturation solubility and dissolution of LF was investigated. Dissolution of marketed formulation and LF-SNEDs was carried out in pH 1.2 and pH 6.8 phosphate buffer.

**Results:**

LF exhibited very high solubility in oleic acid owing to complexation between tertiary amine of LF and carboxyl group of oleic acid (OA). Cremophore EL and medium chain monoglyceride were selected surfactant and co-surfactant, respectively. Significantly smaller droplet size (37 nm), shift in zeta potential from negative to positive value, very high drug loading in lipid based system (> 10%), no precipitation after dissolution are the major distinguish characteristics contributed by LF-OA complex in the SNED system. Saturation solubility and dissolution study in milk containing media pointed the significant increment in solubility of LF in the presence of milk-food fat. LF-SNEDs showed > 90% LF release within 30 min in pH 1.2 while marketed tablet showed almost 0% drug release.

**Conclusion:**

Self nanoemulsification promoting ionic complexation between basic drug and oleic acid hold great promise in enhancing solubility of hydrophobic drugs.

## Introduction

Poor aqueous solubility of the existing and New Chemical Entities adversely affects the oral bioavailability. Failure to mimic in vivo performance compare to in vitro potential, variable absorption and so the plasma concentration, requirement of higher dose than actually needed for desired pharmacological activity are some of the major problems associated with poor solubility of drugs. Further, molecules having very poor aqueous solubility with poor oil solubility impose greater formulation challenges for pharmaceutical scientists. Self emulsifying drug delivery system is one the promising strategy to overcome the solubility barrier of drugs, with commercial products in market e.g. Cyclosporin A, Ritonavir, Lopinavir, Fenofibrate etc. Although a versatile approach, it is not suitable for inherently poor oil soluble molecules e.g. Itraconazole, Carbamazepine, Lumefantrine etc. [1-3].

Lumefantrine (LF) is a highly lipophilic flourene derivative and a Biopharmaceutical Classification System (BCS) Class II drug which is an important agent in the treatment of falciparum malaria. Plasmodium Falciparum is an insidious malarial parasite that fatally threatens a major segment of the Sub-Saharan population in Africa. Thus far, existing therapies for treatment of this form of malaria have been futile due to irregular dosage regimen and insufficient bioavailability afforded by drugs of the quinine class. Lumefantrine is a blood schizonticide, acts by inhibiting detoxification of haem, this toxic haem and free radicals induce parasite death [4,5].

Although a very efficacious molecule, its activity is limited by extremely poor aqueous solubility. Its solubility is far below the critical solubility requirement and so the reported bioavailability is 4–11%. Such vast variability in bioavailability is contributed by the effect of food-fat consumption. Low intrinsic clearance and erratic oral variability and therapeutic levels are more reliably achieved by co-administration with a fatty meal. The oral bioavailability of lumefantrine is highly dependent on food and is consequently poor in acute malaria, showing high degree of variation in different subjects [6]. Poor solubilization leads to incomplete absorption and so inadequate plasma concentrations for antimalarial activity. Due to this chances of treatment failure are higher, which is again associated with increased morbidity, transmissibility and development of resistance. Lumefantrine is an extremely well-tolerated drug, so it is essential to ensure its maximum absorption [6]. Generally milk is recommended to be taken with lumefantrine but availability of milk and its fat content might vary region to region and the variation in antimalarial response to it. This inter-subject variability may gradually induce resistance to artemisinin-based combination therapy, thus making it crucial to increase the dosage regimen. There is only one report on enhancement of dissolution of LF by wet milling technique. However, Nano milling is very high energy consuming process; moreover paper states that nanopowder lumefantrine also requires benzalkonium chloride (BKC) in dissolution media for solubilization [7]. So far there is no report on solubility enhancement of LF by Self nanoemulsifying system. Self nanoemulsifying systems are very well reported in literature for enhancement in solubility of lipophilic drugs. Self emulsifying preconcentrate is made of oil, surfactant, co-surfactant and drug. On dispersion in water it forms < 100 nm sized droplets. Based on oil characteristic it is directly disseminate to systemic circulation or absorb via lymphatic pathway. Oil-surfactant-cosurfactant driven very high solubility, nano-size and permeability results in significantly rise in bioavailability. The spontaneous formation of nanosized emulsion droplets in stomach generates enormously high surface area for drug to diffuse in lumen and absorb rapidly [3,8].

Poor oil solubility of LF has restricted development of lipid based system. In view of this inadequacy, the current study aims at improving the solubility of lumefantrine, especially to eliminate the co administration of milk or any other fatty meal. Considering the basic nature of LF, we have planned to form LF-oleic acid ionic complex and to prepare self emulsifying system of complex by addition of appropriate surfactant. Such a self emulsifying hydrophobic complex enable rapid dissolution of LF, without need of BKC in dissolution media, hence provide better correlation to in vivo condition. Till date, there is no report on preparation of self emulsification system with drug – oil ionic complex. The main objective of the study was to develop a self nanoemulsifying delivery system of lumefantrine to increase its solubility, which otherwise is dependent on food.

## Materials and methods

### Materials

Lumefantrine was procured from Mangalam Laboratories Pvt Ltd (India). The following materials were procured from gattefosse India and were used as received: Labrafac CM10 (C 8 -C 10 polyglycolized glycerides), Maisine 35–1 (glyceryl monolinoleate), Lauroglycol FCC (propylene glycol laurate), Labrafil 1944 CS (apricot kernel oil polyethylene glycol [PEG] 6 esters) and Labrafac PG (propylene glycol caprylate/caprate). Cremophor RH 40 (polyoxyl 40 hydrogenated castor oil), Cremophor EL (polyethoxylated castor oil and Solutol HS 15 (polyoxyethylene esters of 12-hydroxystearic acid) were obtained from BASF India Ltd. Gelucire 44/14 (PEG-32 glyceryl laurate) and 50/13 (PEG-32 glyceryl palmistearate) were received from Colorcon Asia (India). Oleic acid, Tween 80 (polyoxyethylene sorbitan monooleate) and PEG 400 were purchased from Merck (India). Deionized water was prepared by a Milli-Q purifi cation system from Millipore (France). Acetonitrile and methanol used in the present study were of high performance liquid chromatography (HPLC) grade. All other chemicals were reagent grade. Empty HPMC capsule shells were procured from ACG Capsules (Mumbai). Milk of different fat content was purchased from Aarey dairy (1.5% fat content) and Gokul dairy (3% fat content) India.

### Analytical method

A simple HPLC method was developed for quantitative analysis of lumefantrine in the formulation. The HPLC system was equipped with Jasco PU2080 plus pumps with PDA detector and auto sampler unit. The drug was analyzed using Hypersil C18 column (250 mm × 4.6 mm, 5 μm) with mobile phase composition Methanol – 0.1% TFA in water in the ratio of 80:20 v/v, with 1.5 ml/min flow rate and detector wavelength set to 336 nm.

### Methods

#### Screening of oil

Saturation solubility of Lumefantrine in oil was chosen as the criteria of selection. The solubility of the drug was determined in various natural and derived oils. 1 ml of each of the selected vehicles was added to each cap vial containing an excess of LF. Mixing of the systems was performed using a vortex mixer. Formed suspensions were then shaken with a shaker at 37°C for 48 hours. After reaching equilibrium, each vial was centrifuged at 15,000 rpm for 5 minutes. The solubility of lumefantrine in oil was then quantified by HPLC method.

### Screening of surfactant and co-surfactant

Screening of surfactant was done on the basis of (i) Solubility of LF in surfactant solution and (ii) its emulsification efficiency for LF-oil mixture.

Saturation solubility of the drug was determined in various surfactant solution (1% w/v solutions in water) and co-surfactant. An excess amount of LF was added to 5 ml of the surfactant solution and co-surfactant/co-solvent. Samples were placed in a water shaker bath for 48 hrs. The sample was then centrifuged (15,000) for 10 min followed by analysis of supernatant by HPLC. Oleic acid was selected as oil for lumefantrine solubilization. Various Surfactants, co surfactant and combination thereof were mixed with oleic acid and LF-oleic acid solution in various ratios. The co-solvent/co-surfactant were screened on the basis of emulsification time, droplet size, appearance of final system and its reports on compatibility with capsule shell. 500 mg of each mixture (oleic acid-surfactant or LF-oleic acid-surfactant) was added to 250 ml of water (37°C) with mild stirring (100 rpm on magnetic stirrer). The compositions were evaluated for their emulsifying efficiency for oleic acid and LF-oleic acid mixture. Emulsification time, appearance and type of emulsion, LF precipitation and stability for 24 h etc. parameters were considered to evaluate the emulsification efficiency of surfactant.

LF-SNEDs (Lumefantrine-Self Nanoemulsifying Delivery System) was prepared by dissolving LF in oleic acid (minimal amount require for LF solubilization). Optimized mixture of Surfactant and co-surfacatant were added to LF-oleic acid mixture. Fixed weight of Lumefantrine: Oleic acid (100 mg:325 mg) was mixed with various ratios of Cremophore EL and different co-solvents and co-surfactants. Droplet size and emulsification time was evaluated in order to optimize the quantity of surfactant and co solvent/co surfactant. The prepared LF-SNEDs preconcentrate was filled into HPMC capsules.

### Droplet size and zeta potential measurement

One hundred microliters of each LF-SNEDs preconcentrate was added to 100 ml of miliQ water, and gently mixed using a glass rod. The resultant emulsion was analyzed for droplet size (z average diameter) by Dynamic Light Scattering (DLS) using Malvern Zetasizer, USA. The same procedure of dilution used to measure zeta potential by laser dopper microelectrophoresis using same instrument.

### Saturation solubility of lumefantrine in milk containing media

Eventhough LF is recommended to be taken with milk, there has been no literature report hitherto on the effect of milk on the solubility of lumefantrine. In an attempt to check the solubility of lumefantrine in milk containing varying amounts of fat, the following two types of milk were chosen: milk containing 1.5% fat (a) and 3.1% fat (b).

Milk of types a and b were added to different test tubes containing water at pH 1.2 buffer USP (Hydrochloric acid) and pH 6.8 phosphate buffer USP at a concentration of 20% v/v under the assumption that an average person consumes 200 ml of milk in a day. Excess amount of drug was added to each test tube and it was kept in a water shaker bath for 24 hours. Thereafter solutions were filtered through 0.45 μm filter to remove the insoluble drugs. Filtrate was diluted suitably distilled water followed by extracted by chloroform. After evaporating chloroform and reconstituting with mobile phase LF was quantified using HPLC. The saturation solubility of lumefantrine with increasing concentrations of milk at different pH was calculated.

### In vitro dissolution study

Dissolution of Marketed Formulation was carried out in surfactant free dissolution media with and without milk. Instead of using Fed state dissolution media, a real time method to account for variability in ingested food was used by adding 100 mL and 200 mL of low-fat milk (a) to each dissolution flask respectively. The composition of dissolution media for marketed formulation is mentioned in Table 1. Dissolution of marketed preparation was carried out using USP XXIII apparatus I at 37 ± 0.50°C with a rotating speed of 100 rpm. Samples were taken at every 15 min from each of the flasks and the percentage cumulative release was calculated.

**Table 1:** Preparation of dissolution media

**Dissolution media**	**Deionised water**	**Milk**
pH 1.2 buffer USP (HCl)	900	0
	800	100
	700	200
pH 6.8 phosphate buffer USP	900	0
	800	100
	700	200

LF-SNEDs preconcentrate was filled in size ‘0’ HPMC capsules. Dissolution Test of LF-SNEDs was carried out in similar dissolution media using sinker. The composition of milk containing dissolution media showed in Table 1.

## Results

### Screening of oil

The core part of SNEDs is composed of oil, in which drug is solubilized. Hence, it is very much essential to choose the oil having higher solubility for drug. Various types of oil have been screened including fatty acids, medium chain mono/di/tri glycerides, propylene mono/di glycerides and long chain triglycerides. Castor oil and GMO showed minimal solubility of LF while Medium chain triglycerides, Isopropyl myristate, rice germ oil etc. showed moderate solubility of LF (Table 2). The higher solubility in rice germ oil may be attributed to its high oleic acid content and γ-orizynol [9]. Oleic acid showed significantly higher solubility of lumefantrine – 157 mg of LF/gm of oleic acid. Such a higher solubility is not merely expected form hydrophobic interaction between LF and oleic acid. There must be ionic interaction attributes to this solubility enhancement.

**Table 2:** Saturation solubilities of drug in vehicles

**Vehicle**	**Solubility(mg/gm) ± SD(n=3)**
**Oil**	
Castor Oil	5.91± 0.21
Glyceryl Monooleate	7.79 ± 0.29
Sunflower oil	10.57 ± 0.42
Olive oil	11.67 ± 0.37
Acconon CO7	13.22 ± 0.24
Groundnut Oil	14.16 ± 0.43
Corn Oil	19.34 ± 0.61
Captex 300	29.62 ± 0.72
Till oil	33.40 ± 0.8
Isopropyl Myristate	40.85 ± 0.74
Rice germ Oil	59.92 ± 1.19
Oleic Acid	157.20 ± 1.38
**Co**-**surfactant**	
Capmul MCM C8	14.99 ± 0.48
Capmul PG8	18.13 ± 0.49
**Co**-**solvent**	
Propylene Glycol	0.432 ± 0.11
Ethanol	2.831 ± 0.29
PEG 400	2.852 ± 0.18
Transcutol P	19.267 ± 0.58
Benzyl alcohol	78.024 ± 1.41

### Screening of surfactant

Selection of suitable surfactant is very crucial part for self emulsifying system especially when a fine translucent nanosized emulsion is required. Surfactant was selected on the basis of two criterions: saturation solubility of lumefantrine in 1% w/v surfactant solution (Table 3) and its emulsification efficiency for LF-oleic acid (Table 4). Surfactants of chemical diversity – ionic (cholate, SLS) and non ionic (PEG fatty acid esters, PEO-PPO-PEO block co polymers, PEG vitamin E esters etc.) have been screened for solubility of lumefantrine. LF was almost negligible soluble in PEG-medium chain fatty acid ester (Acconon MC8), marginal solubility in ionic surfactant, with highest solubility in Tween 80 (100 ppm). Tween 80 was selected as the surfactant in trials with different co-surfactants to assess the ability of the co-surfactants to improve the clarity of the system. However, Tween 80 does not show good emulsification as the final system remained hazy. Eventhough LF exhibited highest solubility in Tween 80, it was rejected bacause its poor emulsification property for LF-oleic acid (Table 4).

**Table 3:** Saturation solubility of LF in surfactant solution

**Surfactant solution (1%)**	**Solubility (μg/ml) (Mean± SD) (n=3)**
**Acconon MC8**	0.26 ± 0.14
**Sodium Deoxytaurocholate**	1.33 ± 0.09
**Sodium taurocholate**	1.42 ± 0.14
**Tween 20**	9.18 ± 0.13
**SLS**	10.75 ± 0.28
**Lutrol**	13.01 ± 0.15
**Acconon S 35**	13.05 ± 0.17
**Gelusire**	15.18 ± 0.17
**Solutol HS 15**	22.49 ± 0.21
**TPGS**	27.18 ± 0.28
**Cremophore EL**	44.52 ± 0.29
**Cremophore RH40**	46.94 ± 0.25
**Tween 80**	101.63 ± 0.37

**Table 4:** Emulsification behavior of Oil and Surfactant mixture

**Composition**	**Surfactant**	**Observation**	**Emulsification**
**Oleic Acid**	Gelusire 44/14, Tween 80, Solutol HS 15, TPGS, Lutrol F68	Turbid	Poor
**LF**-**Oleic Acid**	Gelusire 44/14, Tween 80, Solutol HS 15, TPGS	Turbid	Poor
**Oleic Acid**	Cremophor RH40	Translucent milky solution	Satisfactory
**LF**-**Oleic acid**	Cremophor RH40	Translucent	Good
**Oleic Acid**	Cremophore EL	Translucent	Good
**LF**-**Oleic Acid**	Cremophore EL	Clear and translucent	Excellent

LF-oleic acid-cremophore EL preconcentrate was self nanoemulsify to 50–100 nm sized droplet depending on the amount of cremophore EL (Table 5). However, in all the bathces have shown longer self emulsification time (~ 7 min) on additon into water (Table 6). Reduction in self emulsification time is necessary to release LF immidiately. In order to reduce the emulsification time, addition of co-solvent or co-surfactant facilitating the emulsification process was added. Various co-solvent/co-surfactant were screened for this purpose. Solubility of LF in Co co-solvent/co-surfactant was not considered as an important criteria for its selection because of very poor solubility of LF in Medium chain monoglycerides, ethanol, PEG and transcutol P. solubiliy of LF was found to be higher in benzyle alcohol compare to other solvents but was rejected in formualtion due to its lower acceptibilty limit and volatile nature.

**Table 5:** Particle size analysis of various formulations

**Formulation**	**Co-solvent/Co- surfactant(mg)**	**Cremophore EL(mg)**	**Particle size(nm)**	**PDI**
F1	0	250	94.51 ± 7.67	0.329
F2	0	325	65.43 ± 5.49	0.229
F3	0	400	48.4 ± 5.3	0.25
F4	Transcutol P (25)	250	72.25 ± 6.7	0.235
F5	Transcutol P (25)	325	60.5 ± 5.3	0.21
F6	Transcutol P (25)	400	50.25 ± 6.2	0.227
F7	Capmul MCM (25)	250	80.39 ± 8.7	0.254
**LF**-**SNEDs**	**Capmul MCM** (**25**)	**325**	**37**.**96** ± **4**.**1**	**0**.**184**
F9	Capmul MCM (25)	400	53.78 ± 4.81	0.211
F10	Capmul MCM (50)	325	39.49 ± 4.4	0.119
F11	PEG 400 (25)	325	52.98 ± 4.7	0.123
F12	Ethanol (25)	325	41.59 ± 3.5	0.139
F13	Capmul PG8	325	51.71 ± 3.9	0.122

**Table 6:** **Effect of different co**-**surfactants on emulsification time**

**Co**-**surfactant**	**Emulsification time(min)**	**Observations**
**Transcutol P**	6.17	Long time to disperse but final system is clear
**PEG**-**400**	4.30	Clear system
**Capmul PG8**	4.70	Translucent nanoemulsion
**Capmul MCM**-**C8**	3.16	Translucent nanoemulsion
**Without any co**-**solvent**	7.0	Highly viscous clumps take a long time to disperse

The emulsification time and appearance of the formulation with different co-surfactants with Cremophore EL were shown in Table 6. Further, droplet size and polydispersity index of various batches of LF-oleic acid mixture with in different ratio of cremophore EL with various Co-surfactant/Co-solvent has mentioned in Table 5.

It was found that a oil:surfactant ratio of 1:1.2 yielded the smallest droplet size and a clear translucent system, however, in an attempt to reduce the amount of surfactant in the system the droplet size was compromised slightly and the surfactant concentration was reduced. Thus, a system with an oil:surfactant ratio of 1:1 was chosen. Although transcutol acts as an effective co-solvent in terms of emulsification capacity, it slows down the emulsification time for the system. PEG-400, inspite of being a good candidate for a co-solvent was not chosen due to its hygroscopic tendencies in soft and hard gelatin capsules. Ethanol was not considered as a co-solvent in the final formulation due to its tendency to diffuse out of the shell and it threatens the integrity of the capsule. Capmul MCM-C8, a medium chain monoglyceride was selected as co-surfactant in finally optimized system (LF-SNEDs) since it has given minimal droplet size of 37 nm (Table 5) with comparatively rapid emulification (Table 6). Moreover there is no report on its any chemical or physical interaction with capsule shell.

### Zeta potential

**Figure 1:**
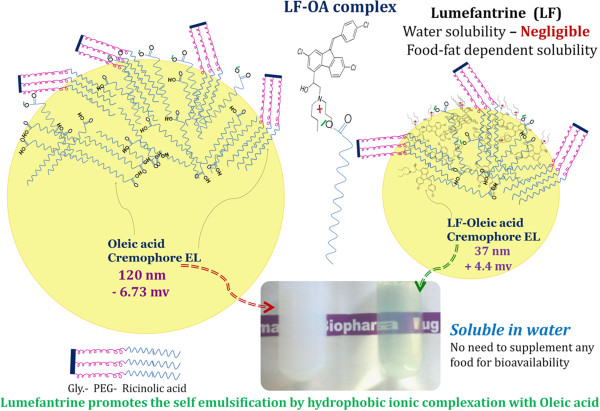
**Graphical presentation of SNED and LF-SNEDs.**

In this study, to account for the electrostatic effects of the drug-lipid interaction, the zeta potential values of self-emulsified formulation were measured at the same drug to lipid ratios as optimized in the above experiments. Zeta potential of SNEDs with and without drug was evaluated to understand effect of LF-oleic acid complex on surface charge. The Zeta Potential of oleic acid self emulsifying system was found to be – 6.73 mv while LF loaded SNEDs exhibited + 4.4 mv zeta potential. The graphical presentation of droplet size, zeta potential and possible orientation of surfactant in LF-SNEDs showed in Figure 1. This indicates the blank formulation has negative zeta potential while addition of drug lead to shift in zeta potential to positive side. The results are in agreement with a study by Nagarsenker et al., suggesting that addition of a basic drug lead to shift in zeta potential from negative to positive [10]. The final composition of LF-SNEDs is mentioned in Table 7.

**Table 7:** **Composition of LF**-**SNEDs**

**Lumefantrine**	**100 mg**
Oleic acid	325 mg
Cremophore EL	325 mg
Capmul MCM	25 mg
Total	775 mg

### Saturation solubility of lumefantrine in milk containing media

**Figure 2:**
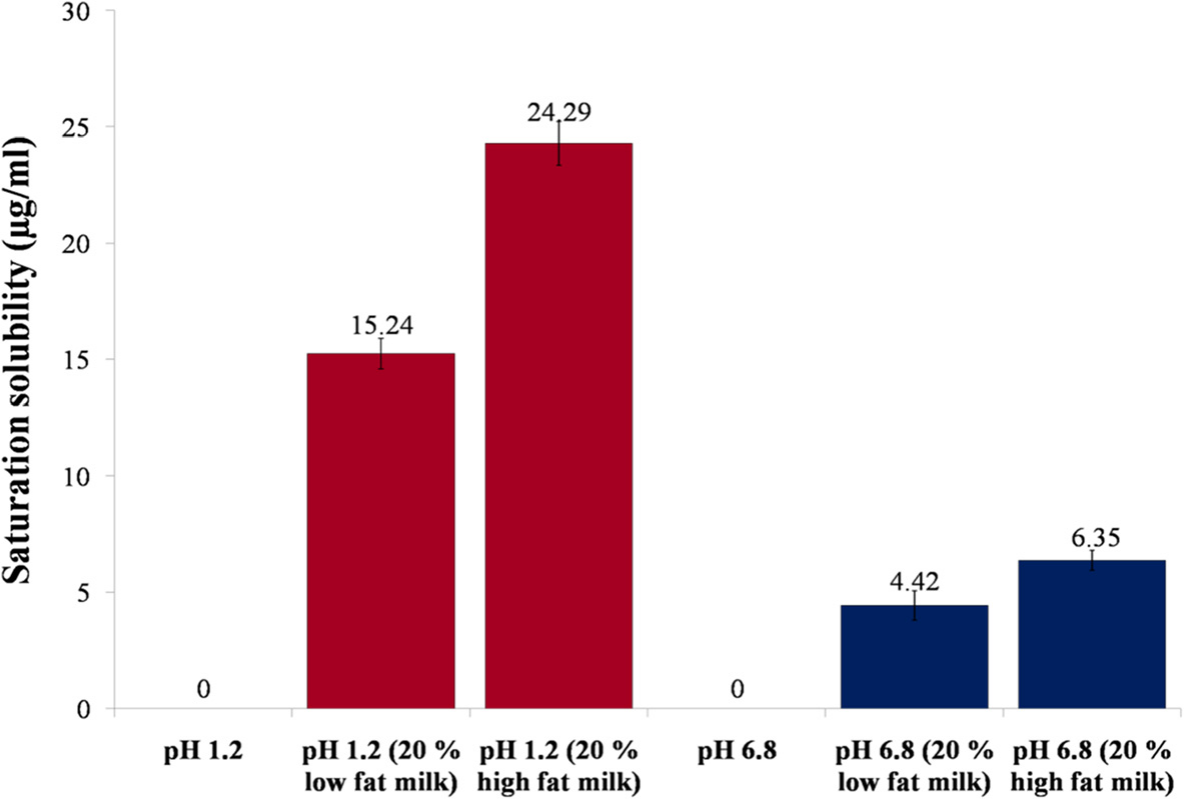
Saturation solubility of lumefantrine in different types of milk at different pH.

The saturation solubility of lumefantrine in milk containing media at gastric and intestinal pH was analyzed (Figure 2). Saturation solubility of LF was found to be significantly influenced by pH and presence milk. However, in absence of milk LF showed almost negligible solubility in both pH 1.2 and pH 6.8 buffers. As expected LF has higher solubility at lower pH due to its basic nature. Saturation solubility of LF increase with increase the fat content of milk, with maximum solubility of 24 ppm was observed in media containing 20% v/v high fat milk at pH 1.2 (Figure 2). Further increase in fat content or milk concentration is expected to proportionally enhance the solubility of LF.

### Dissolution profile of marketed formulation in milk containing media

**Figure 3:**
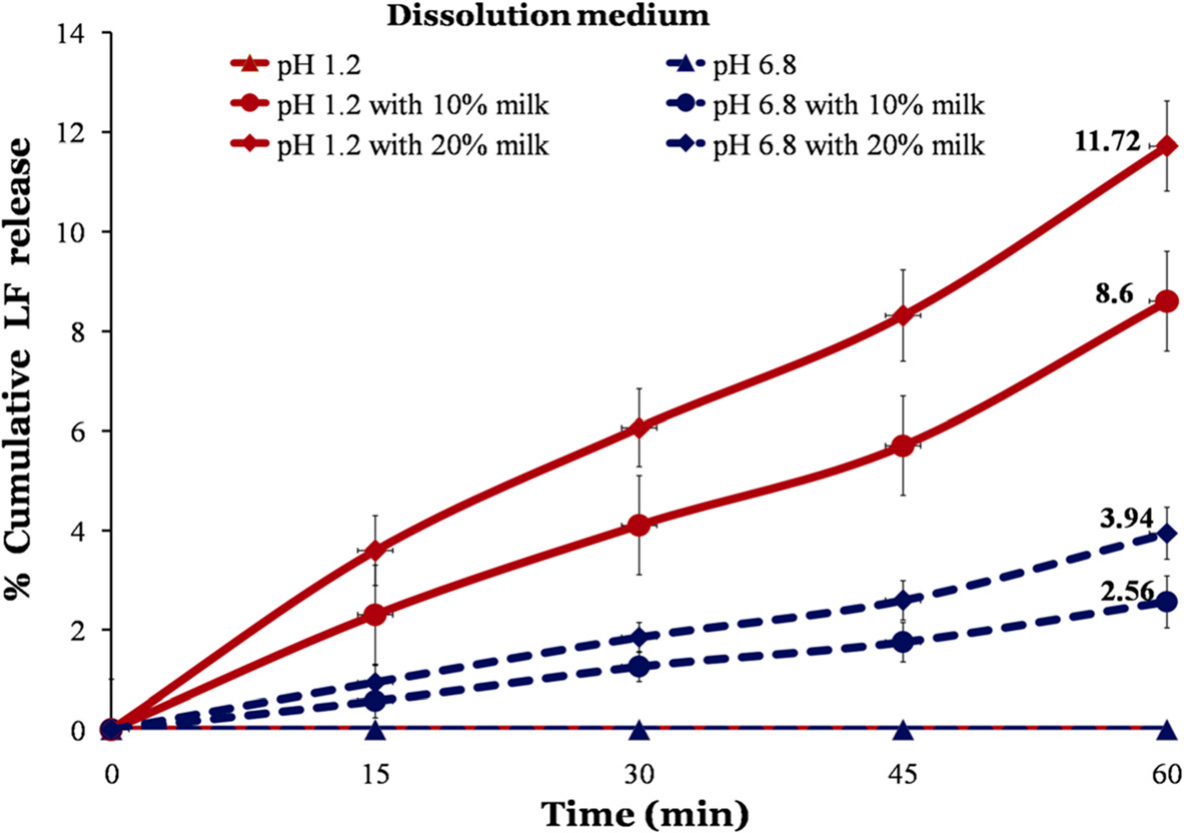
Dissolution profile of marketed formulation in milk containing dissolution media.

Dissolution profile clearly states that release of LF is highly depend on concenration of milk in dissolution media (Figure 3). The results of dissolution sutdies are complemetaty to saturation solubility study of LF in milk containing media. The dissolution medium without milk showed negligible release and hence it can be predicted that without fat containig food suppliment, bioavailbility and therefore therapeutic response may be very poor. Milk containing dissolution media showed marginal improvement in dissolution of LF. Dissolution of LF is higher at pH 1.2 media compare to pH 6.8, irrespective of milk content. Higher dissolution at pH 1.2 is due to its higher solubility at lower pH. The cumulative release increases to maximum 12% upon the ingestion of 200 ml of milk in gastric pH.

### Dissolution test of lumefantrine self nanoemulsifying system

**Figure 4:**
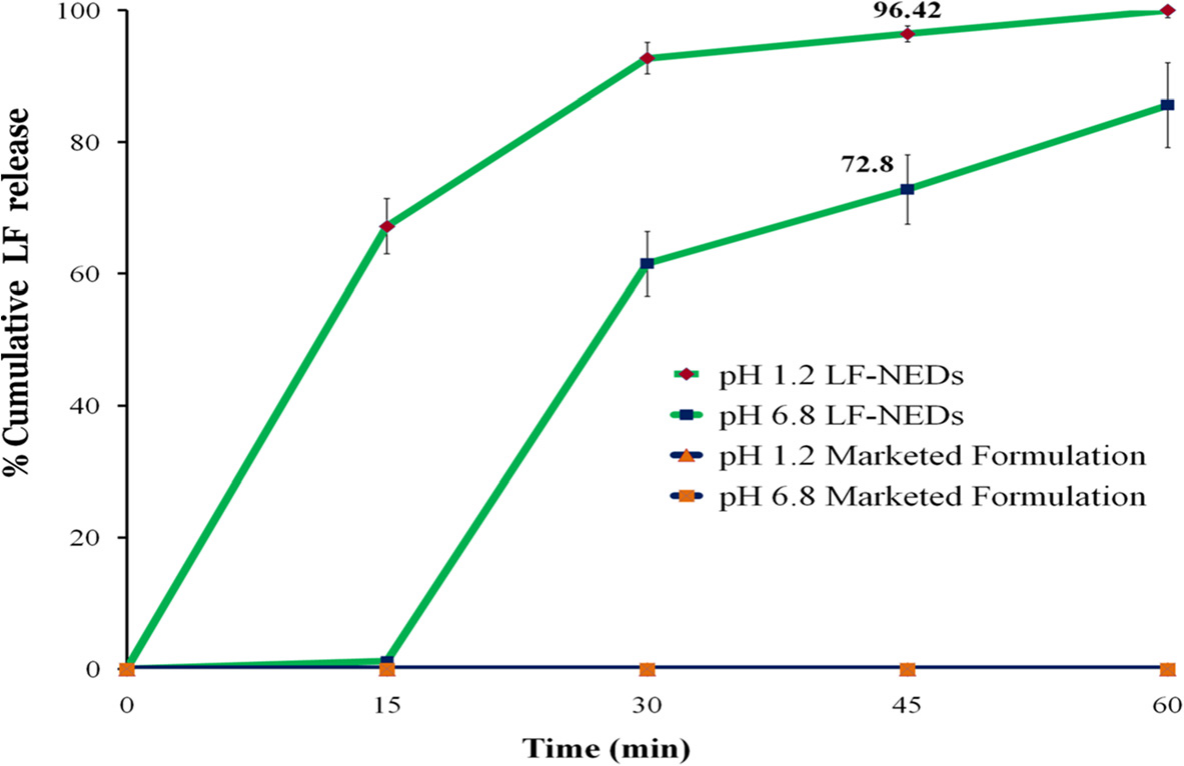
**Dissolution profile of marketed formulation and LF**-**SNEDs.**

Comparable dissoluiton profile of marketed formulation and LF-SNEDs at pH 1.2 and pH 6.8 shown in Figure 4. Marketed formulation showed almost negligible release over the period of 60 min, which is by virtue of extemley poor solubility of lumefantrine in aqeous media. LF-SNEDs exhibited significantly enhancement in dissolution compare to marketed preparation. At pH 1.2, more than 90% of LF was found to release within 30 min account of rapid formation of nanoemulasion in contact with aqeuous medium.

## Discussion

Oleic acid showed highest solubilizaion capacity of LF owing to complexation between tertiary amine of LF and oleic acid. Complexation of LF and oleic acid was indirectly confirmed by addition of stronger base than lumefantrine. It was assumed that addition of stronger amine containing group in oleic acid will interfere in complexation of amine group of LF with carboxylic acid of oleic acid. The reported pKa value of halofantrine (a similar class of drug) is in the range of 8.2 [11]. So on the basis of structural similarity we assumed that lumefantrine has similar pKa. The pKa of Triethylamine (TEA) is 10.5, which depicts that it is stronger base than lumefantrine. LF was found to be insoluble in oleic acid in the presence of TEA. On this basis it was confirmed that ionic complexation is responsible for significant higher solubility of LF in oleic acid.

Further experimentation on self emulsification property of oleic acid and LF-oleic acid suggested the proof of concept of ionic complexation between LF and oleic acid promote self emulsification (see graphical abstract). It was discovered that a system consisting of only oleic acid (no drug), surfactant and co-solvent is self nanoemulsifying to 120 ± 12 nm while system containing LF-oleic acid, surfactant and consurfactant easily emulsify to nano size translucent dispersion of 37.96 ± 4.1 nm. Addition of LF showed 4 times reduction in droplet size. It means that LF-oleic acid complex is itself promoting the self emulsification, which is otherwise difficult to emulsify oleic acid. We can attribute this to the fact that oleic acid interacts with the amine drug and forms a hydrophobic ion-pairing complex with its carboxylic group. Thus, the functional group of oleic acid which might br interfering in self emulsification, on complexation with drug to it promote the self-emulsifying property. Based on the above results, Oleic acid was selected as the oil. The interaction was reflected in zeta potential study. Shift in Zeta potential of plain oleic acid nanoemulsion – 6.73 mv to + 4.4 mv with LF-oleic acid nanoemulsion also support the ionic interaction between amine of LF and carboxylic acid group of oleic acid. The blank formulation has a negative charge due to the predominance of the anionic oleic acid. The negative charge of blank system is due to presence of carboxylic acid group on surface. Very marginal negative potential of the system is due to poor ionization of oleic acid (pKa – 9.85). Moreover, dense network of PEG of cremophore EL on surface mask zeta potential of the ionized species on surface. Zeta potential of LF-SNEDs was found to be slightly positive, clearly indicating the ionic interaction of LF-oleic acid. The positive charge, in LF-SNEDs can be attributed to masking of anionic charge of oleic acid by complexation with LF and surface orientation of amine group of LF in nanoglobules. This interaction results in significantly higher solubility of LF in oleic acid, further LF-oleic acid complex is expected to be more soluble in oleic acid than lumefantrine itself.

Tween and cremphore both are PEG fatty acid esters but their chemical structures have vast diffence. Cremphore surfactants are more bulky and having higher molecular weight compare to Tween surfactants. Tween 80 has single chian of oleic acid as lipophilic part while cremophore surfactants have three fatty acid chain attahced to PEG-glycerol. This bulkier lipophilic part of cremophore may contributed to better emulsification property of cremophore EL and cremophore RH 40. Hence, further formulations were tested with Cremophore EL and Cremophore RH 40. We have observed that free fatty acid e.g. oleic acid is difficult to emulsify in comparision to its glyceryl esters. Though Cremophore RH 40 showed a slightly better solubilising capacity, it was dismissed in favour of Cremophore EL as the latter portrayed a clearer and more transparent emulsion on redispersion. Also, Cremophor RH40 (polyoxyl 40 hydrogenated castor oil) appeared to be less readily digested than Cremophor EL (polyoxyl 35 castor oil). An explanation for differences in the digestability of the structurally similar Cremophor surfactants is not very clear in literature but may reflect differences in the reactivity of the saturated (hydrogenated) castor oil glyceride backbone in Cremophor RH40 leading to the generation of slightly different reaction products with polyethylene oxide, when compared with Cremophor EL (which is generated by polyethoxylation of unsaturated castor oil) [12]. Alternatively the slightly larger polyethylene oxide content of Cremophor RH 40 may have more effectively masked the approach and binding of pancreatic enzymes (and therefore hydrolysis) when compared with Cremophor EL. Cremophore EL has an IIG limit of 599 mg making it a feasible and non-toxic component in the system.

Self-emulsification of oil-surfacatant preconcentrate proceeds through formation of Liquid Crystalline phase (LC) at oil–water interface. The rate and extent of water penetration into LC phase determines the rate of emulsification. Rapidity of self emulsification is governed by weakness and viscosity of intermediate LC [13,14]. Medium chain monoglyceride (MCM) has ability to form such LC phase especially when mixed with hydrophilic surfactant [14].

It was observed that increasing concentrations of fat in milk brought about an increase in saturation solubility of lumefantrine whereas no solubility was observed at the gastric and intestinal pH in the absence of milk. Triglycerides are the major component of milk fat. These medium to long chain triglycerides of milk contributing to marginal solubility of lumefantrine in milk. Higher the fat content of milk, higher will be the solubility of LF in it.

This indicates the extreme necessity of fat containing diet for its solublilization and therefore absoption. Possibility of failure in therapetic response can not be denied with such a poorly soluble drug as discussed in introduction part.

Increase in solubility with increase in milk content is prime reason for enhacement in bioavailability of LF when given with milk. The results are in agreement with a bioavailability study carried out on healthy human volunteer to evaluate the effect of food/fat on bioavailability of LF. Bindschedle et al. have reported 16 fold enhancement in bioavailability in the presence of food [15]. Ashley et al. have reported 90% of maximum AUC was achieved with 36 ml of soya milk [6]. Ensuring that volunteer receives milk or fat with given medicine is feasible under study conditions but difficult to guarantee during routine treatment in malaria patients. However, the availability of milk, composition of fat and the amount of milk consumed varies from person to person and thus there is no conclusive prediction of the bioavailability in the LF.

LF-oleic acid ionic hydrophobic complex emulsify to nanosize by cremophore EL, generating an enormously high surface area. Accroding to noyes-whitney equation reduction in droplet size lead to significant enhancement in dissolution while Prandlt equation suggests the significant reduction in diffusion layer thickness with nanosizing of particle [16].

One more important thing to take into consideration is dissolution media does not contain any surfactant. Generally, in dissolutin studies of hydrophobic drug, surfactant is added to maintain sink condition and to prevent precipitaion of drug-in dissolution media. USP recommonds use of 1% w/v Benzalkonium chloride (BKC) in dissoultion media. The saturation solubilty of LF in 0.1 M HCl with 1% w/v BKC is 119 ± 3 ppm, sufficient to solubilize 120 mg of LF in dissolution media [16]. The most important advantage of LF-SNEDs system is complete dissolution of LF without use of such surfactant in dissoltion media. Both the dissolution media pH 1.2 and pH 6.8, do not contain BKC or other surfactant, still LF-SNEDs capable enough to solubilize drug without precipiatation. Amount of cremophore EL in dosage form is just 325 mg, leading to 0.36% w/v in 900 ml of dissolution media. This concentration is much below to maitain sink condition for LF in dissolution media (Table 3). Hence, we can predict that LF remains in solubilized state in dissolution media because of its comlexation with oleic acid. The complex formation promote the faster self emulsification and dissolution and further inhibit the precipitaion of drug once solubilized. In phosphate buffer 6.8, slow dissolution of capsule shell resulted in 10 min lag period for solubilitzation. After opening of capsule dissolution profile is similar to that of pH 1.2.

## Conclusion

Hydrophobic ionic complexation based self nanoemulsifying delivery system of LF showed remarkabley higher dissolution profile, eliminating the requirement of food/fat for LF solubilization. Lumefantrine has very high solubility in oleic acid due to complexation between tertiary amine of LF and oleic acid. Higher the solubiilty of drug in oil, higher the drug loading capacity of formulation. For drug having higher dose, ionic complexation with oleic acid would be effective strategy to enhance solibilty by self nanoemulisfying formulation. Selection of an ideal surfactant and co-surfactant is very much essential to emulsify the complex to nano sized globule within short period of time. Sponteneous formation of nanoemulsion lead to rapid dissolution of a hydrophobic drug, which may offer food/fat independent bioavailability. Ionic complexation with self emulsifying delivery offer an easy, cost effective and industry feasible approach for solubilization of basic hydrophobic drugs.

## Competing interest

The authors declare that they have no competing interest.

## Authors’ contributions

KP and VS have carried out studies mentioned in article. PV has guided this project and made substantial contributions for data interpretation and involved in drafting the manuscript and revising it critically. All authors read and approved the final manuscript.
